# Sociodemographic and psychosocial risk factors of railway suicide: a mixed-methods study combining data of all suicide decedents in the Netherlands with data from a psychosocial autopsy study

**DOI:** 10.1186/s12889-024-18120-w

**Published:** 2024-02-26

**Authors:** Elias Balt, Saskia Mérelle, Arne Popma, Daan Creemers, Karlijn Heesen, Nikki van Eijk, Isa van den Brand, Renske Gilissen

**Affiliations:** 1 113 Suicide Prevention, Amsterdam, Netherlands; 2 Amsterdam UMC, Amsterdam, Netherlands; 3 GGZ Oost-Brabant, Boekel, Netherlands

**Keywords:** Railway suicide, Prevention, Mixed-methods, Psychosocial autopsy, Risk factors

## Abstract

**Background:**

Railway suicide has profound implications for the victims and their family, and affects train drivers, railway personnel, emergency services and witnesses. To inform a multilevel prevention strategy, more knowledge is required about psychosocial and precipitating risk factors of railway suicide.

**Methods:**

Data from Statistics Netherlands of all suicides between 2017 and 2021 (*n* = 9.241) of whom 986 died by railway suicide and interview data from a psychosocial autopsy of railway suicide decedents (*n* = 39) were integrated. We performed logistic regression analyses to identify sociodemographic predictors of railway suicide compared to other methods of suicide. The Constant Comparative Method was subsequently employed on interview data from the psychosocial autopsy to identify patterns in psychosocial risk factors for railway suicide.

**Results:**

The strongest predictors of railway suicide compared to other suicide methods were young age (< 30 years old), native Dutch, a high educational level, living in a multi-person household (especially living with parents or in an institution), living in a rural area and a high annual household income of > 150.000 euros. Several subgroups emerged in the psychosocial autopsy interviews, which specifically reflect populations at risk of railway suicide. These subgroups were [1] young adult males with autism spectrum disorder who strived for more autonomy and an independent life, [2] young adult females with persistent suicidal thoughts and behaviours, [3] middle-aged males with a persistent mood disorder who lived with family and who faced stressors proximal to the suicide in personal and professional settings, [4] male out-of-the-blue suicides and [5] persons with psychotic symptoms and a rapid deterioration.

**Conclusions:**

based on our findings we propose and discuss several recommendations to prevent railway suicide. We must continue to invest in a safe railway environment by training personnel and installing barriers. Additionally, we should adopt prevention strategies that align the needs of subgroups at increased risk, including young females who have attempted other methods of suicide and young males with autism spectrum disorder. Future research should determine the cost-effectiveness and feasibility of low-maintenance, automated interventions near crossings and psychiatric facilities.

**Supplementary Information:**

The online version contains supplementary material available at 10.1186/s12889-024-18120-w.

## Background

Railway suicide has profound implications for the victims and their friends and family, but also strongly affects train drivers, railway personnel, emergency services and witnesses [1]. Every year, around 1900 people die by suicide in the Netherlands, of which approximately 10% die by railway suicide [2]. The relative incidence of suicide in the Netherlands was 10.6 suicides per 100.000 inhabitants between 2017 and 2021. The proportion of railway suicides was 1.59 suicides per 100.000 inhabitants in males, versus 0.69 in every 100.000 females, and 1.14 in 100.000 in the entire population [2]. On a European scale, suicide and trespassing accidents result in at least 3800 fatalities, representing 88% of all fatalities within the EU railway system [3]. Most victims of railway suicide are male, but the male-female ratio does not differ significantly from other methods of suicide [2, 4]. Concerning however is that young people die more often by railway suicide compared to other methods of suicide. While 15% of the total number of suicide victims in the Netherlands are aged under 30, they represent 31% of railway suicide victims [5]. The same trend can be observed internationally [6].

Research provides insight into why people choose railway suicide as opposed to other suicide methods, but this is mostly based on studies of suicide survivors. Mishara and Bardon [7] reported that individuals who attempted railway suicide were looking for an immediate, certain, and painless death. Easy access has also been suggested as a motivator for railway suicides [8]. Duddin and colleagues [9] presented comparable findings based on suicide notes of people who died by suicide. Decedents perceived railway suicide to be quick and certain. Victims additionally referred to the impersonal nature of railway suicide and discussed their ability to plan the suicide. Marsh and colleagues [10] showed that the planning and preparation of a railway suicide attempt was contingent, and that factors including the likelihood of being seen or interrupted could deter people from a specific method.

Considerable efforts are made to dissuade people from accessing the railway tracks. The strongest lines of evidence point to the effectiveness of safety measures [11–13]. Prevention of railway suicide at locations with elevated risk, or ‘hotspots,’ may be particularly effective to reduce suicides [14–16]. Placing platform screen doors at a station in Japan has reduced the number of railway suicides by 76% [17]. Similar findings are reported in a recent Swedish study, where mid-track fencing at a station reduced suicides there by 62.5%, although there were some concerns about substitution effects, whereby an increase was observed in nearby stations that functioned as a control group [18]. Promising new techniques, including advanced sensor surveillance [19], video monitoring [20] and the effect of lighting [21, 22] have been subjected to theoretical examination and assessed in feasibility studies, but evidence for their effects on a larger scale remains limited.

Some researchers have described demographic risk factors of railway suicide [4, 23, 24], explored individual motivations for railway suicide in victims and attempters [7, 10, 25], identified characteristics of railway suicide locations [14, 16] and emphasized the importance of safety measures [17, 26, 27]. However, to inform a multilevel prevention strategy, more knowledge is required about psychosocial and precipitating risk factors of railway suicide. This would increase our understanding of the suicidal process preceding railway suicide and the needs of individuals at risk. Too and colleagues [28] concluded that *“there is a need for further research activity to strengthen evidence about socio-environmental risk factors for railway suicide”* and suggest that we thereby consider physical, social, and economic factors. We believe this research gap has not yet been sufficiently addressed. Therefore, the objective of this study was to provide recommendations for the prevention of railway suicide by identifying sociodemographic and psychosocial risk factors of railway suicide. Based on former evidence, we hypothesized that railway suicide is more common in young people, and people who used specialized care [23, 29]. No hypotheses were defined for other characteristics like marital status, household income and education level because of limited indications in literature.

## Methods

Nationwide data of Statistics Netherlands were combined with interview data from a psychosocial autopsy study of 39 railway suicide victims in a mixed-methods design. The Medical Research Ethics Committee of the Amsterdam University Medical Centre (NL76295.029.21) approved this study.

### Data selection and study procedures

Non-public microdata of all Dutch inhabitants who died by suicide from January 2017 to December 2021 were extracted from Statistics Netherlands (*n* = 9241) [30]. We primarily used the most recent data available because we aimed to provide recommendations to prevent railway suicide that build on current prevention efforts. In addition, the quantitative data sample was selected from the same time period as the qualitative sample, to facilitate integration of the findings. Coroners of the Community Health Services have labelled the deaths as suicide according to the ICD10 code for death by external trauma: intentional self-harm (X60-X84). The dependent variable, railway suicide, was selected based on the method of suicide (X81). Other methods of suicide represented the reference categories: strangulation and suffocation (X70), medication/alcohol/drugs (X60-X69), jumping from high places (X80), drowning (X71), and other methods of suicide (X72-X79, X82-X84). Sex, age, migration status, education level, marital status, household composition, mental healthcare, general practitioner care, (unemployment) benefits, household income, and urban versus rural residential region were selected as independent variables, guided by the former work of Berkelmans and colleagues [31].

Furthermore, we conducted a psychosocial autopsy. This entailed interviewing proxy informants of railway suicide victims. The psychosocial autopsy was aimed at obtaining deeper insight into the patterns in psychosocial risk factors and proximal precipitating factors of railway suicides. We included suicides from 2017 to 2021. The primary informants of the psychosocial autopsy study were the partners, parents, or children of the victim. A combination of strategies was used to recruit participants, including a brochure for the National Police and the postvention team of the National Railways Services, networks of bereavement support organizations and social media channels of 113 Suicide Prevention. 113 Suicide Prevention is the Dutch helpline for individuals with suicidal thoughts and a leading organization in the field of suicide prevention. We aimed to include secondary informants where possible to triangulate the findings. Secondary informants were selected based on their intensive contact with the decedent in the last months of their lives. A recent Lithuanian psychosocial autopsy by Digryte and colleagues [32] emphasizes the importance of this selection criterium. Secondary respondents included friends, relatives, classmates, or colleagues of the victim. They were recruited through the primary informants. It typically requires approximately twelve homogeneous cases to achieve data saturation in qualitative research [33]. Therefore, we aimed to include bereaved of at least twelve female and twelve male victims, as well as twelve younger victims (≤ 30 years old) and older victims (> 30 years old) to pursue saturation.

Although anecdotal evidence indicates that participation in a psychosocial autopsy is a positive experience, it can be emotionally challenging [34, 35]. Moreover, people bereaved by suicide are at a higher risk to develop suicidal thoughts and engage in suicidal behaviours themselves [36, 37]. Participants were therefore screened for suicidal ideation using the Suicide Ideation Attributes Scale [38] and for symptoms of traumatic grief using an abbreviated version of the Traumatic Grief Inventory [39]. Participants were excluded from the study if they scored above predefined cut-off values. Other exclusion criteria were being a minor (aged < 18), being admitted into a care facility at the time of the study (regardless of psychopathology), the general practitioner advised against participation, and/or the participant did not speak Dutch.

### Data analysis

#### Nationwide statistics data

Age was categorized in ten-year categories 10–20, 20–30, 30–40, 40–50, 60–70 and > 70. Migration background was divided into Dutch natives and people with a migration background. The latter included people who were born outside of the Netherlands, or of whom one or both parents were born outside the Netherlands. Education level was categorized following the Dutch guidelines into low (primary school, secondary school, first three years of college preparatory education), middle (last two to three years of college preparatory education), and high (college or university). Household composition was categorized into living with partner and/or children, living with parent(s), living alone, and other (e.g., inpatient settings). Marital status as single, married, divorced, or widowed. Living area was rural when a person lived in the provinces of Friesland, Groningen, Drenthe, Overijssel, Gelderland, Zeeland, Brabant, and Limburg. Urban living area included the provinces of Noord-Holland, Zuid-Holland, Utrecht, and Flevoland. Specialized mental healthcare was ‘yes’ if reimbursements had been made from healthcare insurance for specialized mental healthcare in the calendar year before the suicide. A limitation is that these healthcare data were not available for people under 18 years old. General practitioner care was ‘yes’ if the reported costs for GP care were higher than the regular registration costs for the GP. Care use was available for people who died between 2017 and 2019. Household income was the gross income in Euros per year for the whole household. These data were available from the years 2017–2020, and divided into five categories: <30.000 euros, 30.000–50.000 euros, 50.000-100.000 euros, 100.000-150.000 euros, and > 150.000 euros. Benefits entailed that a person had received unemployment benefits, welfare benefits, or unfit for work benefits. The variables healthcare and benefit were ‘yes’ if a person received this respective type of support in the period between January 1st of the calendar year preceding the year of the suicide and the time of death. This period thus represents a minimum period of a year and one day, and a maximum of two years. Contingency of the determinants was assessed by calculating Cramer’s V (additional file 1 and additional file 2). The values indicate low to moderate correlation between determinants. No determinants were excluded from analyses.

The number of suicides for sex, methods and year of death was calculated. Subsequently, percentages of subgroups of diverse demographic characteristics were calculated of the total group, per method, and group differences were investigated using Chi-square tests. A *p*-value of < 0.05 was defined as statistically significant. Additionally, multiple logistic regression analysis was performed to determine which demographic characteristics were the strongest predictors of railway suicide compared to other suicide methods. We employed backwards selection to create an adjusted model [40]. All analyses were performed using R (version 4.1.).

#### Interview data

Interviews with the bereaved lasted approximately two-and-a-half hours. The interview instrument was based on former works of international autopsy studies [35, 41–43] and consisted of three parts. Initial questions invited a narrative account of the bereaved about factors contributing to the suicide. In the second part, a reconstruction was made of the last months of the victims’ lives, including the location and circumstances of the suicide, precipitating factors and adverse life events. In the last part, we addressed psychosocial risk factors (additional file 2). The interview topics were informed by empirical evidence about suicide risk factors team [44, 45] and received the consensus of the research team. The instrument has been added as supplement to this manuscript (additional file 3).

Interviews were transcribed verbatim. A team of three researchers coded the interviews, adhering to principles for thematic analyses in qualitative research [46]. First, data were coded deductively using a crude code list based on interview topics. Thereafter, inductive coding was performed to identify emerging themes. We employed the Constant Comparative Method [47]. This entailed that we systematically and iteratively compared the coded data within cases, between cases and between groups (males versus females, victims aged ≤ 30 and > 30) to identify patterns. By patterns we refer to a specific combination of psychosocial factors, precipitating factors and key themes that distinguish a group of individual victims from other cases. These patterns delineate the suicidal process of decedents and highlight needs for care and support.

## Results

### Population statistics

Between 2017 and 2021, 9241 individuals died by suicide in the Netherlands. Most decedents were male (*n* = 6235) and Dutch natives (*n* = 7524). As shown in Table 1 below, 986 individuals died by railway suicide. These were 686 males (70%) and 300 females (30%). Chi-squared analyses reported significant group differences between railway suicides compared to other methods of suicide.

**Table 1:** Characteristics of railway suicide victims compared to other methods

				Total suicides (*N* = 9241)		Railway suicides (*N* = 986)			Other suicides (*N* = 8255)		
				N	%^a^	N	%^a^		N	%^a^	^b^
Sex											
	Male			6235	67.5	686	69.6		5549	67.2	
	Female			3006	32.5	300	30.4		2706	32.8	
Age											
	10–19 years old			316	3.4	82	8.3		234	2.8	***
	20–29 years old			1054	11.4	222	22.5		832	10.1	
	30–39 years old			1166	12.6	133	13.5		1033	12.5	
	40–49 years old			1521	16.5	169	17.1		1352	16.4	
	50–59 years old			2145	23.2	203	20.6		1942	23.5	
	60–69 years old			1532	16.6	109	11.1		1423	17.2	
	70 + years old			1507	16.3	68	6.9		1439	17.4	
Etnicity											
	Dutch			7524	81.4	822	83.4		6702	81.2	
	Migration background			1717	18.6	164	16.6		1553	18.8	
Education level											
	Low			1766	19.1	224	22.7		1542	18.7	***
	Middle			2255	24.4	283	28.7		1972	23.9	
	High			1332	14.4	174	17.6		1158	14.0	
	Unknown			3888	42.1	305	30.9		3583	43.4	
Marital status											
	Single			4205	45.5	587	59.5		3618	43.8	***
	Married			2604	28.2	250	25.4		2354	28.5	
	Divorced			1668	18.0	115	11.7		1553	18.8	
	Widowed			764	8.3	34	3.4		730	8.8	
Household position											
	Child			769	8.3	177	18.0		592	7.2	***
	Couple no children			1963	21.2	171	17.3		1792	21.7	
	Couple with children			1222	13.2	157	15.9		1065	12.9	
	Single no children			4561	49.4	381	38.6		4180	50.6	
	Single with children			307	3.3	33	3.3		274	3.3	
	Other			419	4.5	67	6.8		352	4.3	
Benefits (welfare, unfit for work, or social)											
	Yes			3230	35.0	323	32.8		2907	35.2	
	No			6011	65.0	663	67.2		5348	64.8	
Residential area^c^				*n* = 9100		*n* = 971			*n* = 8129		
	Urban			3867	42.5	341	35.1		3526	43.4	***
	Rural			5233	57.5	630	64.9		4603	56.6	
Household income^c^				*n* = 7171		*n* = 784			*n* = 6387		
	< 30.000			2490	34.7	257	32.8		2233	35.0	***
	30.000–50.000			1584	22.1	125	15.9		1459	22.8	
	50.000-100.000			1921	26.8	215	27.4		1706	26.7	
	100.000-150.000			782	10.9	115	14.7		667	10.4	
	> 150,000			394	5.5	72	9.2		322	5.0	
Healthcare use GP in 1 to 2 calendar years before suicide^c^				*n* = 5457		*n* = 601			*n* = 4856		
	Yes			3453	63.3	351	58.4		3102	63.9	**
	No			2004	36.7	250	41.6		1754	36.1	
Healthcare use specialized in 1 to 2 calendar years before suicide^c^				*n* = 5459		*n* = 601			*n* = 4858		
	Yes			2007	36.8	240	39.9		1767	36.4	
	No			3452	63.2	361	60.1		3091	63.6	

*a. Due to rounding off, percentages may not add up to exactly 100%*

b. Chi-squared test for group differences, *p < 0.05, ** p < 0.01, *** p < 0.001

*c. For these determinants, data was not available for the years 2020 and/or 2021*

The multiple logistic regression model in Table 2 presents sociodemographic factors associated with railway suicide compared to other methods of suicide. The adjusted model includes the strongest predictors of railway suicide. Decedents of railway suicide were more often aged < 20 (OR: 3.56, CI: 2.31–5.49 ) or 20–30 year old (OR: 2.37, CI: 1.80–3.14), lived in a rural area (OR: 1.47, CI: 1.25–1.73), were Dutch natives (OR of migrant versus Dutch: 0.78, CI: 0.62–0.96), had a higher education level (OR: 1.58, CI: 1.20–2.10), lived in a household composition of more than one person, such as with parents (OR: 1.53, CI: 1.13–2.07), with a partner (OR: 1.28, CI: 1.01–1.62), with a partner and children (OR: 1.50, CI: 1.16–1.95) or an institutional household (OR: 2.20, CI: 1.55–3.06), and had a high household income (OR: 1.43, CI: 1.05–1.94).

**Table 2:** Multiple logistic regression model (crude & final)

Railway suicide (*n* = 986) versus other suicide methods (*n* = 8255)							
		Crude model^a^			Adjusted model^b^		
		OR	95%CI	^c^	OR	95%CI	^c^
Sex (ref: male)							
	Female	1.08	0.88–1.31				
Age (ref: 50–59)							
	10–19 years old	3.98	2.32–6.80	***	3.56	2.31–5.49	***
	20–29 years old	2.60	1.84–3.68	***	2.37	1.80–3.14	***
	30–39 years old	1.33	0.95–1.84		1.31	1.00-1.72	*
	40–49 years old	1.11	0.83–1.48		1.14	0.89–1.46	
	60–69 years old	0.81	0.58–1.11		0.75	0.57–0.99	*
	70 + years old	0.45	0.28–0.70	***	0.46	0.32–0.65	***
Rural area (ref: urban area)							
	Yes	1.50	1.24–1.82	***	1.47	1.25–1.73	***
Migration background (ref: Dutch)							
	Migration background	0.77	0.60–0.99	*	0.78	0.62–0.96	*
Education level (ref: low)							
	Middle	1.44	1.09–1.91	*	1.31	1.03–1.67	*
	High	1.78	1.27–2.49	***	1.58	1.20–2.10	**
	Unknown	1.35	1.00-1.83	*	1.24	0.96–1.61	
Household position (ref: single no children)							
	Child	1.39	0.97–1.99		1.53	1.13–2.07	**
	Couple no children	1.21	0.87–1.66		1.28	1.01–1.62	*
	Couple with children	1.38	0.95–2.01		1.50	1.16–1.95	**
	Single with children	1.27	0.75–2.04		1.39	0.90–2.09	
	Other	1.92	1.25–2.88	**	2.20	1.55–3.06	***
Income (ref: 50.000-100.000)							
	< 30.000	1.25	0.96–1.64		1.15	0.92–1.44	
	30.000–50.000	1.00	0.75–1.32		0.90	0.70–1.15	
	100.000-150.000	1.15	0.85–1.55		1.11	0.86–1.43	
	> 150.000	1.56	1.08–2.24	*	1.43	1.05–1.94	*
Marital status (ref: single)							
	Married	0.98	0.70–1.37				
	Divorced	1.00	0.73–1.34				
	Widowed	0.88	0.51–1.46				
Healthcare use GP in 1 to 2 calendar years before suicide (ref: no)							
	Yes	0.89	0.73–1.08				
Healthcare use specialized in 1 to 2 calendar years before suicide (ref: no)							
	Yes	1.15	0.94–1.41				
Benefits (welfare, unfit for work, or social) (ref: no)							
	Yes	0.87	0.69–1.08				

*a. Crude multivariate model*

*b. Adjusted model with strongest predictors of railway suicide after backwards selection*

c. *p < 0.05, ** p < 0.01, *** p < 0.001

### Psychosocial risk factors for railway suicide

Fifty-six bereaved individuals registered for the interview, of whom fourteen withdrew participation (nonresponse or informed withdrawal) and two were excluded. One participant was excluded because there was already a primary informant involved for the decedent, and one was excluded because it did not involve a railway suicide. In total, thirty-nine cases of railway suicide have thus been studied in the psychosocial autopsy study. In seventeen cases, a secondary informant was interviewed. In other cases, the primary respondent did not refer the researchers to a secondary informant during the study period. The suicide victims were thirty males and nine females, aged 14 to 75 years old. Table 3 summarizes the demographic characteristics of the sample population.

**Table 3:** Psychosocial autopsy sample

	Respondents (*n* = 56)
**Participants (interviews)**	
Interview with partners	10
Interview with parents	20
Interview with children	4
Interview with peers, siblings	18
Interview with other informants (E.g., healthcare professional)	4
	**Cases (** *n* ** = 39)** ^**a, b**^
**Sex, n (%)**	
Male	30 (77%)
Female	9 (23%)
**Age, mean (SD)**	36.4 (15.7)
Aged < 30, n (%)	16 (41%)
Aged 30≥, n (%)	23 (59%)
**Ethnicity, n (%)**	
Native Dutch	33 (85%)
Migration background	6 (15%)
**Region of the suicide, n (%)**	
Rural	23 (59%)
Urban	16 (41%)

^a^. Due to rounding off, percentages may not add up to exactly 100%

^b^. Categories with less than five observations were merged

### Location of the suicide and circumstances of death

Most victims (*n* = 31) died along the railway tracks, with no train station in proximity, of whom 23 near a crossing. A much smaller group (*n* = 8) accessed the tracks from station platforms. The locations of railway suicides were primarily selected for proximity and familiarity. Based on the reports, victims travelled 15 to 20 min on average to the location of the suicide. Locations were commonly frequented in the victims’ daily lives, for example when commuting to work or school. Less reported factors were that the location had personal meaning to the victim and that the location was remote and quiet.

Two individuals stood out because they lived close to the tracks but travelled far to the location of the suicide. Particularly notable was a girl who suffered from severe anxiety and had not left her house in months. On the day of her suicide, she uncharacteristically travelled for two hours across the country to an unfamiliar location. Her brother noted that she had searched for suitable locations online, and that this location had a history of railway suicide fatalities.

Sibling: *“I was surprised, because I thought: if she will do it, it’ll be near [town]. She lived five minutes away from the railways, but she took the bus to get to a place of which the technical forensics department knew it frequently came up for railway suicides, because it was a “successful” place.”*

Respondents stated that the primary motivation for railway suicide as a method was the victims’ perception of a quick, painless, and effective way to end their life. Other motivations included that railway suicide was seen as impersonal (compared to other methods), that their loved one wanted to die away from their home and next-of-kin, or that they were afraid of other methods of suicide. Seven respondents did not know why the victim chose railway suicide specifically.

### Preparation of a railway suicide

The suicide was known to be prepared by 35 of the 39 victims. Preparation comprised multiple actions over a period ranging from weeks to several months. Although individuals prepared the suicide in their own way, the sequence of preparation events was typically consistent. A prototypical scenario was that a victim first sought information about suicide or suicide methods online. Information seeking was frequently discovered post-mortem in the online browser history of the victim. After collecting information, decedents scouted potential locations for the suicide. Several decedents then planned a date and time when they wanted to die. One notable example describes a young boy who told his parents about a school assignment during the COVID-19 lockdown for which he needed to be at school. It turned out that there was no assignment and that this was the planned date for his suicide.

Parent: *“He did everything through Zoom. I asked him: ‘how do things work right now?’*

Same as before,’ he replied. ‘I have to go to school on [date] for a project.’ […] He must have made it up. He left his laptop at home. He just took a sandwich with him. […] turns out there was no school project.

In the weeks before the suicide, preparations concerned practical matters, such as cancelling subscriptions, settling financial matters, or writing a will. Interpersonal preparation events happened proximal to the suicide and included saying goodbye to loved ones and writing a farewell note.

### Psychosocial and precipitating factors of railway suicide: pattern analysis

In this study, we set out to combine findings from population data and a psychosocial autopsy. We identified five patterns in psychosocial and proximal precipitating factors of the studied suicides, which distinguished subgroups of decedents compared to the other cases. These patterns are crude and explicitly not meant to be interpreted as exclusive typologies of suicide. Not all cases in our study fit one of the identified patterns. We instead describe them to contextualize psychosocial and precipitating factors of the suicide in the victims’ lives, and highlight needs for care and support congruent with the problems that they faced. Three of the identified patterns aligned with the two strongest identified predictors of railway suicide, namely young age and multi-person or institutional households. These patterns thus provide context to the outcome of our regression analyses.

#### Pattern 1: entrenched

Adverse life events in the last months preceding the suicide relating to work and romantic relations were explicitly associated with the suicide of nine working males aged between 37 and 58 years old. These men were furthermore characterized by recurrent moderate to severe mood disorders, with episodes up to two decades before the suicide. Six males received specialized mental healthcare (therapeutical and/or medicinal) at the time of death.

Proximal stressors in the personal and work spheres, including pressing responsibilities at work and problems with colleagues, (fear of) job loss, relationship problems, or an overall perceived inadequacy made it increasingly difficult for them to stay positive about the future and increased their distress over the last months of their lives. Feelings of anxiety were often reported to aggravate the already present depressive thoughts. Employers were unaware of the problem’s magnitude, partners of the men explained. The men only truly expressed their negative thoughts and feelings towards their partners.

Respondents reflected on the growing support needs of their respective partners and fathers, and the profound strain this put on the family setting, particularly where children were involved. Crippled by the situation, the men’s partners sought ways to live with the situation. To illustrate, the partner of one man who had frequent emotional breakdowns introduced a daily moment for him to vent his troubles, away from their children.

Partner: *“I felt I had to be strict to protect the children. So, whenever he suddenly started crying, or fell on his knees, I would say: ‘not here,’ you know? ‘If you truly feel so horrible, you should go upstairs and close the door for a moment.’”*.

#### Pattern 2: autism spectrum disorder and a struggle for autonomy

In seven young males aged 14 to 32, various parallels presented themselves in psychosocial and precipitating factors of the suicide. Three of them lived at home, one was admitted in a care facility, and three lived on their own or with roommates. Notably, six of them were diagnosed with or suspected of having autism spectrum disorder (ASD). The youths were described by next-of-kin as normative or stern, and their lack of resilience was emphasized. Respondents associated these traits with a predisposition for railway suicide, being “orderly” and “dependable.”

A theme that clearly stood out was the desire to obtain a stronger sense of autonomy in their lives, for example by landing a well-paid job or finding an apartment. They became disillusioned when they encountered their own limitations and felt they had no prospect of self-sustenance and independence. To cope with these feelings, the young males engaged in risk behaviours, explained respondents. Six started consuming alcohol or drugs, of whom five developed an addiction.

Parent: *“three years earlier, he also had a period during which he struggled. He smoked marijuana. And made debts. And then, for a month or so, he got treatment in specialized mental healthcare, and he lived at home for some nine months. […] He re-enrolled in his education, and he found a job. And we thought: things are looking up. That’s what you want for your child. […] What really tore at him was that things didn’t work out again. Because, in the end, he started smoking pot again. And he got into debt again. Even though these were problems that could be fixed.”*

The last months of their lives were marked by increased feelings of frustration and externalizing behaviours. Respondents recalled incidents of verbal and physical aggression, problems with social relations, financial problems, and relapse into substance use addiction. One young man lost his job, and another called in sick in the month before the suicide. Four others experienced school problems, of whom three dropped out. Despite their problems, they often had no history of suicidal behaviour. Only one of the young men had survived an earlier suicide attempt. Suicide-related communication was limited. While their behaviours reflected their struggles, the young men never explicitly mentioned having suicidal thoughts.

#### Pattern 3: persistent suicidal thoughts and behaviours

For a group of seven young females and one young male, aged between 16 and 31 years old, the process towards the suicide was described as a gradual, downward spiral. They were diagnosed with psychiatric problems in early adolescence, most commonly a personality disorder, mood disorder, behavioural disorder, eating disorder, or a combination of these. Respondents explained how the youth’s psychiatric problems were a key contributing factor for the suicide. Other salient themes in the interviews aside from persistent psychopathology were the respondents’ perceptions about healthcare, specifically continuity of care and treatment perspective, frequent suicide-related communication, and persistent and increasing suicidal behaviours.

The suicidal process covered years and described deliberate self-harm and suicide-related communications from the onset of psychiatric problems in early adolescence. Over time, self-harming behaviours became more frequent and suicide-related communication more explicit.

Parent: *“everyone knew [about her suicidal thoughts]. It was all she ever talked about in the end. […] No, except for the first times, we weren’t taken aback by it anymore. It became like a status [to talk about suicide].”*

Six of the seven decedents had engaged in multiple suicide attempts, whereby it stood out that they used progressively lethal methods. Respondents suggest that from the perspective of these young people, railway suicide was a “*final resort*,” because it represented *“surety.”*

Although the victims had explored a plethora of treatment options, it was hard to establish their care needs because of psychiatric comorbidity. Consequently, they were sent from one location to another and struggled to rebuild their trust in a new practitioner. The problems worsened for several years, by which time they lost perspective and hope of future improvement.

Parent: *“You learn to deal with it. […] We actually didn’t expect that she would live to be 16 years old. I just hoped… if she would make it through puberty, then we could have accompanied her to a euthanasia clinic, and we could have let her go peacefully in her sleep.”*

#### Pattern 4: psychotic symptoms

Six victims experienced a rapid deterioration of their mental health following a psychotic episode. The decedents had diverse demographic characteristics. Three were aged under 30, and three aged 30 years or older. What connected them was the presentation of psychopathology in the last months prior to the suicide. Two of them were diagnosed with schizophrenia. However, psychotic symptoms, ranging from paranoid thoughts to visual and auditory hallucinations, were reported as influential to the suicide of all six cases.

Central themes were the disruptive effects of acute psychotic symptoms on professional help and support, the rapid increase of distress preceding the suicide, and the focus on acute treatment (medicinal treatment of psychotic symptoms) as opposed to addressing underlying problems. All six victims received medicinal treatment in the months before they died, some of whom had just started. Next-of-kin felt that the focus on symptomatology was driven by necessity and note that a sustainable way to help their loved ones had not yet been worked out.

Parent: *“[He was at the door and] I saw he was a little anxious, panicky. He had told us earlier that he sometimes heard voices. I said: ‘come in, what happened?’ He had seen someone in his garden. […] up until today we still do not know if this was true or if he hallucinated, saw images. He called the Police, that much has been confirmed. […] he told his story to me. I saw doubt in his face. He even said: ‘mom, I’m not actually sure it was real.’ I asked him: ‘did you bring your meds? So, he took a pill and went to sleep.”*

#### Pattern 5: out-of-the-blue suicides

Finally, the suicide of nine decedents was described as unexpected, or *out-of-the-blue*. The perception of the respondent was leading in this classification. The decedents were eight males and one female. Four were aged under 30, and five were aged 30 and up. Frequently mentioned psychosocial characteristics were mild to no psychiatric problems, the absence of suicide-related communications, and no history of suicidal behaviour. Care seeking behaviours and healthcare use were limited.

Because there were few signs of distress, respondents were unable to explain why their loved ones had died by suicide. By contrast to other cases, where psychopathology was consistently linked to the suicide, an innate emotional vulnerability was instead emphasized in out-of-the-blue cases. The victims were described as sensitive, introverted, insecure, or sub-assertive. They appeared resilient enough to cope with negative thoughts and feelings at the time. However, in retrospect, next-of-kin attributed the death to a combination of psycho-emotional vulnerability and accumulating psychosocial stressors.

Parent: *“What was decisive… I really can’t say. But I think that dormant problems; a study that wouldn’t work out, never having had a relation, no perspective of having a relationship. […] Really just because… there were so little things that were going well for him.”*

## Discussion

The aim of this study has been to identify sociodemographic and psychosocial characteristics of railway suicide victims. Almost a thousand people died by railway suicide between 2017 and 2021 in the Netherlands. We hypothesized that, compared to other methods of suicide, railway suicide was more common among young people and people who used specialized healthcare. Regression analysis showed that being young, of Dutch origin, having a high education level, living in multi-person households, living in rural areas and having an annual household income of > 150.000 euros were significant predictors of railway suicide compared to other methods of suicide. The psychosocial autopsy elucidated the process leading up to a railway suicide, which often included notable stressors and life events in the months prior to the suicide and a thorough preparation of the suicide. The interviews indicated several subgroups of people who may be more prone to railway suicide.

People in the age categories of 10–20 years old (OR: 3.57) and 20–30 years old (OR: 2.37) had increased odds to die by railway suicide. These findings are in line with earlier research [4, 6]. Interventions to prevent railway suicide of young adults specifically should be considered, using information that visually and linguistically appeals to young adults. An inspiring example is the Australian Rail Safety Week [48]. Two subgroups of young adults emerged in the psychosocial autopsy. For one group of young adult females, railway suicide represented a last resort after a prolonged process of persistent suicidal thoughts and increasingly lethal suicidal behaviours. They received extensive care, but the effects of treatment were hampered by psychiatric comorbidity. Van de Koppel and colleagues [49] have well addressed the needs of young women with chronic suicidality based on earlier autopsy studies, including autonomy-promoting treatment policy, treating suicidality as a transdiagnostic phenomenon, and creating a multidisciplinary network of care providers, which may effectively deter these young people from railway suicide. Another group of vulnerable young adults concerned males with developmental disorders, particularly ASD, who struggled for autonomy. The young males were further characterised by increasing psychosocial problems and externalizing behaviours, including verbal and physical aggression and substance abuse. Based on experts’ suggestions, these young males would strongly benefit from interventions appealing to their communication and problem-solving skills [50–52]. When these interventions are organized with a focus on societal inclusion, for example in the context of school and work, we may prevent them from dropping out. Respondents suggested that young males with ASD may have a predisposition for railway suicide, because it aligns their lines of thought. There is indeed some evidence that adolescents with high functioning ASD more often engage in lethal suicide attempts, including on the railways, compared to other adolescents with suicidal ideation [53] and an in-depth case study by Mikami [54] suggests that they may be predisposed to (attempt to) die by railway suicide due to personal cognitions such as a love for trains, travel and order.

People living in the ‘other’ household (which includes institutionalization) had high odds to die by railway suicide compared to other methods. It is known that the proximity of a psychiatric hospital is a risk factor for railway suicide [4, 15]. Overall, multi-person households had increased odds of dying by railway suicide compared to other methods, with odds ratios ranging from 1.28 to 2.20 compared to single people living alone. Decedents had wanted to die somewhere away from family and friends, and preferably without bystanders. People living with family may therefore have higher odds to die by railway suicide, as these circumstances are not met in their home environment. One of the subgroups identified in the psychosocial autopsy consisted of middle-aged males (40–70 years old) living with family. These men were unable to balance their perceived responsibilities as a parent or partner with their mental health problems. The researchers identified signs of psychological entrapment following an accumulation of psychosocial stressors in the months preceding the suicide [55]. Contrary to indications in literature suggesting that males are less likely to seek care for mental health problems [56], most males in our sample received psychiatric care for mood disorders. However, considering the psychosocial nature of their problems, re-envisioning psychiatric care as a touchpoint for psychosocial support may have merit to prevent suicide in this group. Additionally, support for the family of males with persistent mood disorder can help alleviate the burden within the family setting.

Other determinants had a small effect size. Living in a rural area increased the odds to die by railway suicide (OR: 1.47). Internationally, a high track-density, inherent to urban areas, has been associated with increased railway suicides [7]. In the Netherlands, track density is high throughout the country, which could mitigate this effect [57]. Alternatively, the psychosocial autopsy indicates that decedents selected a secluded location near their home and that they knew, like a crossing with little traffic, and such locations may be more readily found in sparsely populated regions. Lastly, two socioeconomical determinants, a high education level (OR: 1.58) and the highest income category (OR: 1.44), were associated with railway suicide. This suggests that people with a higher socioeconomic status are more likely to die by railway suicide, but there is no clear theoretical basis in literature, so this requires further enquiry.

### Limitations

This study has several limitations. Firstly, there were some limitations to the national data. Education level was unknown for many cases. Moreover, information about specialized care use was not available for individuals aged under 18 years old and we did not have data on healthcare use in 2020 and 2021. Recent changes in healthcare provision may therefore not be reflected in our findings. Nonetheless, the data concern all suicide victims in the Netherlands, which enabled a robust investigation of sociodemographic risk factors. Secondly, we aimed to include 80 cases of railway suicide in the psychosocial autopsy to allow statistical testing of dichotomous variables. The recruitment procedure of autopsy studies is notoriously time and resource intensive [35]. We included 39 cases of railway suicide instead of the estimated 80 cases. Another important limitation of the psychosocial autopsy study is that no control group was included. Therefore, we cannot be sure if the themes and patterns addressed in the autopsy are specific to railway suicides. Considering the parallels in themes identified in earlier autopsy studies [35, 58] we believe that sociodemographic factors noted in our study also apply to other suicide methods. Nevertheless, we believe these patterns reflect the needs for care and support of subpopulations at risk, particularly by having experts and professionals translate the findings into meaningful treatment and prevention strategies [49, 59]. Lastly, our psychosocial autopsy findings may not be culturally sensitive due to challenges with the inclusion of cases with a migration background in the interviews. 18.8% of all suicides in the Netherlands have a migrant background. Although for railway suicides this number is lower (16.6%, CBS microdata), we believe that alternatives for prevention are required because people with a migrant background tend to seek professional support for mental health problems less than their native counterparts, and various other barriers prevent them from using care resources, such as difficulties with (medical) language, diversity of needs, and personal convictions and beliefs [60].

## Conclusions and implications

This study has been among the first to integrate findings from population statistics and psychosocial autopsy data to obtain a deeper understanding of the sociodemographic, precipitating and psychosocial risk factors of railway suicides. Railway suicides were often prepared, and a typical sequence of preparative events was identified. Importantly, this represents a window of opportunity for prevention. We must continue our efforts to invest in a safe railway environment, training railway personnel to recognize signs that reflect preparation and installing barriers to prevent railway suicide. Additionally, future research should investigate the cost-effectiveness and feasibility of automated, low-maintenance interventions such as information signs or blue lights, particularly in proximity to crossings and psychiatric care facilities. Conceivably, these interventions may also deter individuals who are contemplating or preparing a railway suicide. Several subgroups emerged in the psychosocial autopsy. Interventions that appreciate the needs of these subgroups are commendable, including a better integration of psychiatric care and psychosocial support for middle-aged males, a multidisciplinary network approach in mental healthcare for young adult females with persistent psychiatric problems, and comprehensive programmes to better support young males with autism spectrum disorder in schools.

### Electronic supplementary material

Below is the link to the electronic supplementary material.

Supplementary Material 1

Supplementary Material 2

Supplementary Material 3

## Data Availability

Interview data cannot be shared publicly because of ethical restrictions: the dataset contains potentially identifying and sensitive information and the Medical Research Ethics Committee (MREC) of Amsterdam UMC has imposed this restriction (registration number: NL76295.029.21). Contact: metc@vumc.nl, https://www.vumc.nl/research/overzicht/medisch-ethische-toetsingscommissie. Data may be made available upon reasonable request. Contact the author (EB) for enquiries.
