# Supplementary figures and images for: Sociodemographic and psychosocial risk factors of railway suicide: a mixed-methods study combining data of all suicide decedents in the Netherlands with data from a psychosocial autopsy study
Source: BMC Public Health. 2024 Feb 26;24:607. doi: 10.1186/s12889-024-18120-w (PMC10895750; doi:10.1186/s12889-024-18120-w)

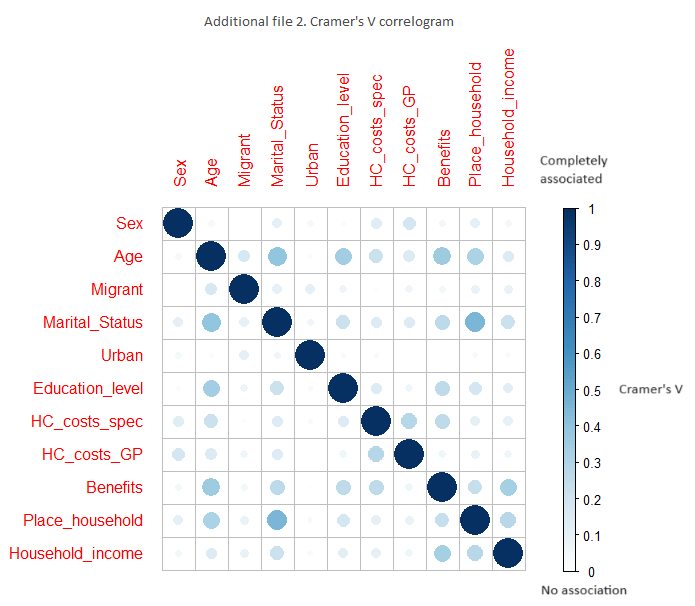

Supplement: Supplementary file 2 — Supplementary Material 2 [file 12889_2024_18120_MOESM2_ESM.tif]
