# Supplementary material for: Sociodemographic and psychosocial risk factors of railway suicide: a mixed-methods study combining data of all suicide decedents in the Netherlands with data from a psychosocial autopsy study
Source: BMC Public Health. 2024 Feb 26;24:607. doi: 10.1186/s12889-024-18120-w (PMC10895750; doi:10.1186/s12889-024-18120-w)
Supplement: Supplementary file 3 — Supplementary Material 3 [file 12889_2024_18120_MOESM3_ESM.pdf]

Additional file 3. Interview instrument for psychosocial autopsy after railway suicides

Definitive version - approved by MREC (Medical Research Ethics Committee) of the Amsterdam University Medical Center

Amsterdam, the Netherlands, 2021

Considerations for the interviewer

The goal of the interview is to learn from suicides on the railway. With the information from the interviews, we aim to develop railway suicide prevention strategies in collaboration with, among others, NS/ProRail and the National Police.

When probing follow-up questions, ensure that you deepen rather than broaden. This means that you do not ask for more examples but seek clarification on how this factor contributed to the suicide. In-depth follow-up questions aim to uncover what prompts a bereaved person to mention a specific factor. Standard questions include: What did that look like? What impact did it have? In what way did it play a role in their (thoughts of) suicide?

It is crucial not to impart any sense of guilt for the death. Family members and survivors may feel guilt, and this should not be reinforced. All aspects associated with the death should be discussed tactfully.

Bereaved individuals may become upset and should be given the necessary time to talk when they wish to do so. When they are less distressed, ask if the interview may continue. It is also possible that family members express a lot of anger or resentment, for example, towards the healthcare system. There should be room for this, but these feelings should not be reinforced or contradicted.

It is desirable that all items on the interview list are addressed. It is, however, possible to deviate from the order of questions to allow the conversation to flow naturally. Note: In some conversations, bereaved individuals may answer a question in their narrative before it is formally asked. In such cases, you may skip that question.

The interview should not commence until the interviewee has given informed consent. Informed consent for the audio recording of the interview must also be requested and signed. Enter the conversation fit and well-prepared. Take care of your emotional safety/individual aftercare in consultation with the interview coordinator at 113.

Before the interview

- Introduce the interviewer and researcher. Provide a brief impression of yourself, establish personal contact. Describe the roles you have within this research and the interview.
- State the purpose of the research. We aim to learn from the suicide of your deceased partner/child/family member/friend/colleague/student. With the information gathered from the interviews, we intend to develop, where possible, suicide prevention strategies in collaboration with organizations such as NS/ProRail and the National Police.
- Discuss privacy: This conversation is confidential, and the data will be processed anonymously. Only the team of the directly involved researcher has access to the data.
- The purpose of this interview is to gain insight into what preceded the suicide of [...], and what you believe played a significant role leading up to this suicide.
- We want to learn from every suicide; our goal is not to investigate who or what is to blame for the suicide. Suicide is complex, but we aim to better understand it.
- Based on our experience, we understand that it can be challenging to examine the factors leading to the suicide of [...]. The questioning may give the impression that you could have prevented it, but that is not the intention; we want to carefully explore together whether there are lessons to be learned from this suicide.

Practical

- There will be at least one break, and more if you wish.
- You can stop the interview at any time.
- I understand that discussing the death of [...] can evoke strong emotions. We will start with questions about the months leading up to the death. These questions also relate to the suicide method and the day of the suicide, and they can therefore be particularly heavy. You can press the 'pause button' whenever you feel it is too much. At that moment, we will assess together if and how we can proceed.
- This part of the conversation will be led by me. [NAME OF INTERVIEWER 2] will record your answers and may occasionally interrupt to clarify something.
- The part about the last months is followed by an interview section where we want to examine factors that played a role, like problems at work or in healthcare. To learn as much as possible from suicides, it is important that we can compare the outcomes effectively. In the second part, questions are often asked in a closed format, meaning the answer is yes or no. If you want to expand because you feel something is a crucial aspect there is, of course, room for that. The questions in this part will be asked by [NAME INTERVIEWER 2].

After the interview

- Thank the participants for their story and their participation in the research.

- Ensure that the participants have all the information they need. Let them know they can have access to the data.
- After the interview, check if there are any unmet needs among the bereaved. Discuss the possibilities for follow-up care if it seems necessary and feasible, preferably with their family doctor.
- Write a brief (half-page) report on how the interview went. It should focus not on the content but on an impression of the well-being and emotional situation of the participants during the interview (before, during, and after).

0. Prior information

Information provided by the interview coordinator

0.1 Interview date: ...

0.2 Respondent code: ...

0.3 Gender of the decedent:

- Male
- Female
- Other

0.4 Date of Birth: ...

0.5 Age: ...

0.6 Place of Residence: ...

0.7 Postal Code (4 digits): ...

0.8 Did [name] reside in this residential area at the time of the suicide?

0.8.1 If not, where did [name] stay?

0.9 Province:

- Groningen
- Friesland
- Drenthe
- Overijssel
- Flevoland
- Gelderland
- Utrecht
- Noord-Holland
- Zuid-Holland
- Zeeland
- Noord-Brabant
- Limburg

0.10 May we see a photo of [...]?

0.11 Depending on the relationship between the respondent and the deceased, the following questions are formulated to get to know each other a bit better before the reconstruction.

0.11.1 What did the daily life of [...] look like (within the family)?
0.11.2 How did you meet [...]?
0.11.3 What was your relationship with [...] like?

0.12 What is the reason for your participation in this research?

Part I – A reconstruction of the last month

In this part of the interview, we would like to focus on the last month of [....] live. Details can be valuable to us. You are the one who knew [....]. You can tell us everything you remember and noticed.

Last month

1.1 We would firstly like to ask you if you can briefly describe what [....]'s life was like in the last month before their passing? Please tell us everything that comes to mind, even things you think might not be important. Take your time and follow your own pace.

1.2 Did [...] have suicidal thoughts in the last month?

1.3 How was your contact with [....] in the last month of their life?

1.4 How often did you have contact with [....] during that period?

- Never
- Occasionally
- Regularly
- Often
- Very often

Last contact

1.5 When was the last time you had contact with [....]?

1.5.1 What did that contact look like?

1.5.2 How did [....] behave during that last contact?

1.5.3 What did [....] look like during that last contact? Did anything stand out?

Last day(s) alive

1.6 Do you know what mood [....] was in on the day of their passing?

1.7 Did their mood change during the last three days before their passing? It is okay if you do not know. If you were not there, you might have heard about how they were doing on the last day.

1.7.1 If yes, to what extent? (improved/not improved/no change)

1.8 Did [....] express feelings of hopelessness?

1.8.1 Can you provide an example?

1.9 Could [....] still look to the future with a positive outlook?

1.9.1 Can you provide an example?

1.10 Did [....]'s behavior change during the last three days before their passing? What did that look like?

Noteworthy events/adverse life events last month and last day

1.11 Did something happen in the month before [....]'s passing that could have contributed to the suicide?

1.11.1 If yes, what happened?

1.11.2 If yes, when did the event occur?

1.11.3 If yes, how did [....] react to these event(s)?

1.11.4 If yes, did they talk to someone about it?

1.11.5 What was said at that time?

1.12 Did something happen on the day of their suicide that could have played a role in the suicide?

1.12.1 If yes, what happened?

1.12.2 If yes, how did [....] react to these event(s)?

1.12.3 If yes, did they talk to someone about it?

1.12.4 What was said at that time?

1. **Location, Preparation, and Exploration Date and Time**

2.1 What was the date of the suicide?
2.2 What was the time of death? It is okay if you do not know the exact time at which the suicide took place.

Location

2.3 Are you familiar with the location along the railway where [...] lost their life?

2.4 Do you know what this location looks like?

2.4.1 If yes, can you describe it for us? All details can be valuable for learning purposes.

2.4.2 Do you know if [....] was familiar with the location where he/she ended their life?

2.4.3 How far from home was it? (ask for an estimated distance in kilometers if possible)

2.4.4 Was it along the track, or at a station/platform? Do you know which platform?

2.4.5 Did [name] pass away due to collision with a high-speed train or a local train?

2.4.6 Did [name] stand, sit, lie down, or took another position?

2.4.7 Was the suicide near a level crossing?

2.4.8 Was it a rural or remote area, or was it close to a residential area or traffic hub?

2.4.9 For instance, was it a quiet place or busy?

2.4.10 Was it sheltered or open? For example, many trees, bushes, or an open field?

2.4.11 Was it a place with many lights? For example, streetlamps, lights from surrounding houses.

Exploration and Preparation

2.5 Did you notice anything in [....]'s behavior that gave you the impression they were preparing for suicide?

2.6 How did [...] prepare for the suicide?

2.7 What specific behavior do you remember that made you think of suicide preparation?

2.8. Did [....]'s train travel behavior change in the last month?

2.8.1 Did [...] go to places near the railway more often in the last month?

2.8.2 Was [...] away from home more often than usual, cycling, walking, or driving around the area?

2.8.3 Did [....] search for information on suicide methods on the internet?

2.9 When did [...] do this?

2.10 How often did it occur? ...

- Never
- Occasionally
- Regularly
- Often
- Very often

2.11 Was alcohol and/or medication/drug intake confirmed at the time of death (excluding death by overdose)?

2.11.1 If yes, which substances were taken? It is okay if you do not know the exact substances

2.12 Was the deceased alone at the time of suicide?

2.12.1 If not, who was with them?

2.13 Did [...] contact a support organization or 113 Suicide Prevention on the day of death, as far as you know?

2.14 Do you know why [...] specifically chose to end their life by suicide with a train?

2. Other Preparations

Expressions or signs of preparation in the last month

3.1 Did [....] say anything in the last month that gave you the impression they were planning suicide? This could also be to someone other than yourself (e.g., saying goodbye).

3.1.1 If yes, to whom did they express this?

3.1.2 If yes, do you know when this took place?

3.1.3 If yes, what was said?

3.1.4 If yes, what was the reaction of that person?

3.2 Has [...] ever written something that gave the impression they were planning suicide?

3.3 Has [...] ever shared anything on social media or elsewhere on the internet that gave the impression they were planning suicide?

Farewell Letter

3.4 Was a farewell letter or another object found after the death?

3.4.1 If yes, who was the message addressed to?

3.4.2 If yes, does the letter provide a reason for the suicide?

3.4.3 If yes, what is that reason?

3.4.4 If yes, what other information could be found?

3.4.5 If yes, was there a mention of possible mental health issues in the message?

3.4.6 If yes, is there an opportunity to see the farewell letter/object? If yes, do you agree to allow us to photograph this letter/object? We understand this is very personal information and want to emphasize that you are free to refuse this request.

3.5 Do you think something could have been done to prevent the death of [...]? What do you think would have helped [...]?

3.6 What is a beautiful memory you have of [...]?

Part II – Open narrative question

Thank you for everything you have shared about [...].

We will soon begin the part of the interview in which we examine factors that played a role in the death of [...]. In this part, [INTERVIEWER 2] will ask the questions and enter the answers into the system. You will notice that there are more closed questions in this section, but there is always room for you to share more if you wish. Before we discuss factors contributing to the suicide of [...], I would like to invite you to briefly reflect on what you think played a significant role in the suicide of [...]

Narrative – contributing factors

Looking back on [...]'s life, which factors or events do you think have significantly contributed to the suicide of [...]?*

*Note to interviewer – this question can trigger a lot of different topics and extensive information. It is important to ask follow-up questions that explore the topics in depth, and not in breadth. That means that you should not probe for other themes, but explore how the presented themes played a role to the suicide. For example, if a person had problems at work, what did these problems look like? How did the person deal with them? How were they associated with their feelings of distress and possible suicidal thoughts and/or behaviours?

Additionally, this question is the bridge to the third part. Once you have discussed the presented contributing factors, note that some parts will be further discussed in the next session. This can help you to adhere to the schedule. Try to limit the time for this narrative to approximately 15 minutes.

Part III – screening of psychosocial risk factors

PERSONALITY

We would like to get an understanding of the personality of [...].

1.1 How would you describe the character of [...]?

1.2 Can you provide an example of how you observed this in [...]? ...

1.3 Do you believe the personality of [...] played a role in the suicide?

- Yes
- No
- I don't know

1.4 If yes, would you like to elaborate? ...

MARITAL STATUS AND CHILDREN

2.1 What was the marital status of [...]?

- Married/registered partnership
- Cohabiting
- Unmarried, never married
- Divorced, living separately
- Widow, widower

2.2 With whom did [...] live? Multiple answers are possible.

- With a partner/spouse
- With child(ren) under 18 years
- With child(ren) 18 years and older
- With his/her parent(s)
- With another adult/other adults
- Did not live with a partner but had a long-term relationship
- Was living alone

With a partner

2.3 How long was [...] together with their partner? ...

2.4 How would you describe their relationship? Was it a stable relationship? ...

No partner

2.5 How did [...] feel about being single? ...

2.6 Did [...] experience a relationship breakup in the past year?

- Yes
- No
- I don't know

2.6.1 If yes, what was the impact on him/her? ...

2.7 Do you believe that a broken relation or other relationship problems played a role in the suicide?

- Yes
- No
- I don't know
- N.A., [...] had no relational problems

2.7.1 If yes, would you like to elaborate? ...

Children

2.8 How many children did [...] have? How old are the children? ... (0 or go to question 2)

2.9 Were there any issues with the children of [...]? (health, relational, school-related, etc.)?

- Yes
- No
- I don't know

2.10 Do you believe that problems concerning the children of [...] played a role in the suicide?

2.11 if yes, would you like to elaborate?

3. Migration background

3.1 In which country was [...] born?

☐ Netherlands

☐ Suriname

☐ Dutch Antilles

☐ Turkey

☐ Morocco

☐ Poland

☐ In another country, namely...

☐ I don't want to say

☐ I don't know

3.2 In which country was [...]’s mother born?

☐ Netherlands

☐ Suriname

☐ Dutch Antilles

☐ Turkey

☐ Morocco

☐ Poland

☐ In another country, namely...

☐ I don't want to say

☐ I don't know

3.3 In which country was [...]’s father born?

☐ Netherlands

☐ Suriname

☐ Dutch Antilles

☐ Turkey

☐ Morocco

☐ Poland

☐ In another country, namely...

☐ I don't want to say

☐ I don't know

3.4 What language did [...] speak at home?

3.5 Has [...]'s experience with their migration background played a role in the suicide?

□ Yes

□ No

□ I don't know

□ I don't want to say

□ N.A., [...], was of Dutch origin

3.6 If yes, would you like to elaborate? ...

4. Work

4.1 What was the highest level of education attained by [...]

□ Lower General Secondary Education (Mavo/VMBO)

□ Higher General Secondary Education (Havo)

□ Pre-University Education (VWO, Gymnasium)

□ Intermediate Vocational Education (MBO)

□ Higher Vocational Education (HBO)

□ University

□ Other, namely...

4.2 Did [...]'s encounter significant problems with reading or writing? For example, illiteracy, dyslexia, or dyscalculia.

□ Yes

□ No

□ I don't know

4.3 If yes, what kind of problems? How did this affect him/her? ...

4.4 Was [...]'s IQ tested?

□ Yes

□ No

□ I don't know

4.4.1 What score resulted from the test?

4.3 Was [...]'s employed at the time of death?

 Yes

 No, [...] was unemployed

Working

4.3 What was [...]'s occupation at the time of death?

4.4 How long had [...] been working there?

4.5 Did [...] find pleasure in their work? (found work interesting, enjoyable, educational, important, engaged)

4.6 Were there any changes at work before the suicide?

 Yes

 No

 I don't know

4.7 Were there any conflicts at work with colleagues, supervisors, clients, etc.?

 Yes

 No

 I don't know

4.8 Did [...] take sick leave in the year before their death?

 Yes

 No

 I don't know

4.9 Do you think there is something related to work or previous work situations that influenced the suicide?

☐ Yes

☐ No

☐ I don't know

4.10 If yes, would you like to elaborate? ...

Unemployment

4.11 How long was [...] unemployed?

4.12 What was the reason for the unemployment?

4.13 How did [...] feel about being unemployed?

4.14 Do you think the unemployment was related to the suicide?

☐ Yes

☐ No

☐ I don't know

4.15 If yes, would you like to elaborate? ...

5. FINANCIAL SITUATION

5.1 What was the financial situation of [...]? (income regular and sufficient, struggling to make ends meet) ...

5.2 Were there financial problems?

☐ Yes

☐ No

☐ I don't know

5.3 Did [...] have debts?

☐ Yes

☐ No

☐ I don't know

5.3.1 If yes, can you say something about the extent and nature of those debts? (For example, gambling debts, forced home sale) ...

5.4 Did [...] deal with bailiffs and/or collection agencies?

☐ Yes

☐ No

☐ I don't know

5.5 Did [...] attempt to seek help for these problems?

☐ Yes

☐ No

☐ I don't know

5.5.1 If yes, how did [...] try to resolve the problems? (bank, loan, debt restructuring) ...

5.6 Did [...] anticipate possible future financial problems?

☐ Yes

☐ No

☐ I don't know

5.7 Do you think the financial problems of [...] influenced the suicide?

☐ Yes

☐ No

☐ I don't know

☐ N.A., [...] did not have any financial problems

5.8 If yes, would you like to elaborate? ...

6. SOCIAL RELATIONS

6.1 Did [...] have social relationships (offline/online)?

 Yes

 No

 I don't know

6.1.1 With whom? (family, friends, colleagues, neighbors, etc.) ...

6.1.2 Roughly estimated, how many of these people would [...]'s considered as a good friend?

6.1.3 How often did [...]'s have contact with these people? never occasionally regularly

 often

 very often

6.2 Did [...] feel sufficiently supported (practically/emotionally) by friends?

 Yes

 No

 I don't know

6.3 Did [...] feel that they could be supportive (practically/emotionally) to friends?

□ Yes

□ No

□ I don't know

6.3 Were there any problems/conflicts with friends in the year before the death?

□ Yes

□ No

□ I don't know

6.4 Do you think problems with social relationships influenced the suicide?

□ Yes

□ No

□ I don't know

□ N.A. There were no problems with social relationships

6.5 If yes, would you like to elaborate? ...

7. SOCIAL MEDIA, GAMES, AND SERIES

7.1 Did [...] experience anything on social media that may have influenced the suicide?

□ Yes

☐ No

☐ I don't know

☐ N.A., [...] rarely or never used social media

7.2 If yes, would you like to elaborate? ...

7.3 Did [...] experience anything in games that may have influenced the suicide?

☐ Yes

☐ No

☐ I don't know

☐ N.A., [...] rarely or never played games

7.4 If yes, would you like to elaborate? ...

7.5 Did [...] see anything in online series, e.g., Netflix, that may have influenced the suicide?

☐ Yes

☐ No

☐ I don't know

☐ N.A., [...] rarely or never watched online series

7.6 If yes, would you like to elaborate? ...

8. EXAMPLES OF SUICIDALITY IN ENVIRONMENT

8.1 Has anyone in the family of [...] attempted suicide?

☐ Yes

☐ No

☐ I don't know

8.2 Has anyone in the family of [...] died by suicide?

☐ Yes

☐ No

☐ I don't know

8.2.1 If yes, when and which family member(s)? ...

8.3 Has any friend or acquaintances of [...] attempted suicide?

☐ Yes

☐ No

☐ I don't know

8.4 Has any friend or acquaintances of [...] died by suicide?

☐ Yes

☐ No

☐ I don't know

8.4.1 If yes, when and which friend(s) or acquaintance(s)? ...

8.5 Was [...] exposed to the suicide or attempted suicide of a person in the media briefly before their own suicide?

☐ Yes

☐ No

☐ I don't know

8.6 Do you think any of these experiences could have influenced the suicide?

 Yes

 No

 I don't know

 N.A., [...], did not know anyone in the environment with (attempts at) suicide

8.7 If yes, would you like to provide more details? ...

!

SUICIDAL BEHAVIOR

9.1 Has [...] ever harmed themselves on purpose, for example by cutting themselves, or by hitting their head or hands hard against a wall or other object?

- Yes
- No
- I don't know

9.1.1 If yes, how often? ...

- Never
- Occasionally
- Regularly
- Often
- Very often

9.2 Has [...] ever attempted suicide before? (or refer to earlier attempts based on reconstruction)

- Yes
- No
- I don't know

We would like to work with you to understand the attempts made by [...].

9.3 When was the first suicide attempt by [...]?

9.4 How did [...] try to end their life at that time?

- Poisoning
- Hanging
- Drowning
- Cutting
- Jumping from a height
- Jumping in front of a moving vehicle
- Burning
- Other, namely...

9.5 [Repeat: questions about attempts between the first and last attempt; document the order of methods and timeline]

9.6 In what way did [...] try to end their life in the last non-lethal suicide attempt?

- Poisoning
- Hanging
- Drowning
- Cutting
- Jumping from a height
- Jumping in front of a moving vehicle
- Burning
- Other, namely...

9.7 When was this last, non-fatal attempt?

10. SUICIDAL THOUGHTS AND SUICIDE-RELATED COMMUNICATION

10.1 What was the first indication, if any, that [...] was thinking about suicide? This can be something you noticed in their behaviour, but also something that they said or had written.

10.1.1 When did you notice this? ...

10.2 Did [...] ever talk about not wanting to be alive anymore or about wanting to die, with you or anyone else?

- ☐ Yes
- ☐ No
- ☐ I don't know

10.2.1 If yes, with whom? What was said at that time? ...

10.3 How often did [...] talk about death or his/her suicidal thoughts?

- ☐ Never
- ☐ Occasionally
- ☐ Regularly
- ☐ Often
- ☐ Very often

10.4 Was [...] preoccupied with death? (for example, a notable interest in dying or suicide in the media)

- ☐ Yes
- ☐ No
- ☐ I don't know

EUTHANASIA

10.5 Has [...] ever discussed euthanasia?

- ☐ Yes
- ☐ No

□ I don't know

10.6 If yes, had [...] initiated a procedure for euthanasia? ...

BARRIERS TO SUICIDE-RELATED COMMUNICATION, NON-DISCLOSURE

If there has been no discussion at all about the decedent’s suicidal feelings, inquire as follows:

10.7 You have indicated that [...] never expressed his/her suicidal thoughts or feelings. Do you have an idea of what prevented [...] from talking about this?

11. PHYSICAL PROBLEMS

11.1 Has [...] been diagnosed with a physical problem or disease?

This also includes diseases that were managed through treatment.

□ Yes

□ No

□ I don't know

11.2 If yes, would you like to elaborate? ...

11.3 Did [...] have a physical disability?

□ Yes

□ No

□ I don't know

11.4 Can you tell us what physical disability this was? ...

11.5 Did [...] suffer from pain?

□ Yes

□ No

□ I don't know

11.6 If yes, would you like to elaborate? ...

11.7 Did [...] use prescribed medication for a physical disease?

□ Yes

□ No

□ I don't know

11.8 If yes, how did [...] comply with these medications? Did [...] follow the prescribed doses?

11.9 Do you think physical problems have played a role in the suicide?

□ Yes

□ No

□ I don't know

11.10 If yes, would you like to elaborate? ...

12. Mental health problems

12.1 Did [...] have mental health problems or psychiatric disorder?

□ Yes

□ No

12.2 If yes, had a diagnosis been established? (multiple answers are possible)

□ Schizophrenia/psychotic disorder

□ Bipolar mood disorder

□ Depressive mood disorder

□ Social anxiety disorder/generalized anxiety disorder/phobia

□ Obsessive-compulsive disorder (OCD)

☐ Eating disorder

☐ Alcohol addiction or abuse

☐ Drug addiction or abuse

☐ Personality disorder:

- Antisocial
- Borderline
- Avoidant
- Dependent
- Obsessive-compulsive
- Narcissistic
- Other, ...
- […] only had traits of the following personality disorder…

☐ Somatoform disorder

☐ Post-traumatic stress disorder

☐ Attachment disorder

☐ Attention deficit hyperactivity disorder

☐ Oppositional defiant behavior disorder

☐ Autism spectrum disorder

☐ Adjustment disorder

☐ Another disorder, ...

12.3 If yes, would you like to provide more details? (when was the disorder diagnosed, any suspected diagnoses not yet established, any primary diagnosis in case of multiple diagnoses)…

11.3 Did […] have an intellectual disability?

☐ Yes

☐ No

□ I don't know

12.4 Do you know which intellectual disability this was?... (for example, a mild intellectual disability)

12.4 Did anyone in the family of [...] have a psychiatric illness or mental disorder?

□ Yes

□ No

□ I don't know

12.4.1 If yes, what diagnoses? ...

12.5 When did the mental health problems of [...]

□ Less than 12 months ago

□ 1-2 years ago

□ 2-5 years ago

□ More than 5 years ago

□ No mental disorder

12.6 How did [...] cope with their mental health problems?

12.7 How did [...] perceive their mental health problems to affect them in the future? Did they their situation would improve? ...

12.8 Did […] experience trauma in their life?

 Yes

 No

 I don't know

12.9 If yes, what trauma did […] experience? (e.g., physical abuse, sexual abuse, emotional abuse, cyberbullying) …

12.10 Do you think mental health problems and/or traumas played a role in the suicide?

 Yes

 No

 I don't know

 NA, […] did not have any mental health problems or trauma

12.11 If yes, would you like to elaborate? …

13. TREATMENT

13.1 Did […] receive one or more of the following forms of care? (multiple answers are possible)
 Psychiatric care, mental health care (outpatient)
 Psychiatric care, mental health care (inpatient)
 General practitioner, GP Psychologist
 Other care, namely: …

13.2 Was […] prescribed medication for a psychiatric disorder?
 Yes
 No

13.2.1 If yes,
 What medications were those? ...
 How long did [...] use these medications? ...
 What was the purpose of these medications? ...
 What was the result of the medications? ...
 Was [...] increasing or decreasing their dosage at the time of death? Or were they receiving a fixed doses? ...
 Had [...] recently changed their medication at the time of the suicide?

13.3 Did [...]
 Yes
 No

13.3.1. If yes,
 What treatment/therapy was that? ...
 How long did [...] follow this treatment? ...
 What was the purpose of this treatment/therapy? ...
 What was the result of this treatment/therapy? ...

13.4 Was there a focus on suicidal thoughts and/or behaviours in the treatment of [...]?
 Yes
 No
 I don't know

13.4.1 Would you like to elaborate? ...

13.5 Was [...] treated at the time of the suicide?
 Yes
 No
 I don't know

13.6 Was [...] admitted into a psychiatric hospital at the time of the suicide?
 Yes
 No
 I don't know

13.7 By which institution/organization/person was the treatment provided at the time of death? (general practitioner, psychologist, psychiatrist, social worker, psychiatric hospital) ...

13.8 When was the last appointment with the therapist? .....

13.9 During the last appointment, did [...]
 Yes

☐ No
☐ I don't know

13.91. Would you like to elaborate? ...

13.10 Was [...] on a waitlist for mental healthcare at the time of the suicide?
☐ Yes
☐ No
☐ I don't know

Could you tell us what it was for and how long: ...

13.11 Had [...] just finished a treatment in mental healthcare at the time of the suicide?
☐ Yes
☐ No
☐ I don't know

13.12 Was [...] treated by other professionals, such as social workers or a local coach?
☐ Yes
☐ No
☐ I don't know

Would you like to elaborate? ...

13.13 Have you, as a close relation, received treatment or guidance?
☐ Yes
☐ No

13.14 What treatment/guidance was that? ...

13.15 How long did this treatment/guidance last? ...

13.16 What was the result of this treatment/guidance? ...

13.17 Was [....] familiar with the Dutch Suicide Helpline (113 Suicide Prevention)?
☐ Yes
☐ No
☐ I don't know

13.18 If yes, do you know if [....] used the helpline?
☐ Yes
☐ No
☐ I don't know

13.19 If yes, what were his/her experiences with the helpline of 113 Suicide Prevention? ...

13.20 Do you think that [...] has had any experiences with mental healthcare that have played a role in the suicide?
☐ Yes
☐ No
☐ I don't know
☐ N.A., [....] did not receive any healthcare

13.21 If yes, would you like to elaborate? ...

13.22 Did [...] have any unmet mental healthcare needs that have played a role in the suicide?
☐ Yes
☐ No
☐ I don't know
☐ N.A., [....] did not receive any healthcare

13.23 If yes, would you like to elaborate? ...

14. ALCOHOL

14.1 Did [....] regularly drink alcoholic beverages?

☐ Yes

☐ No

☐ I don't know

14.1.1 If yes, what types of alcoholic beverages? How often? In what quantity? ...

14.2 If yes, when [....] drank alcohol, did he/she drink excessively?

☐ Yes

☐ No

☐ I don't know

14.3 Do you think [....]'s alcohol consumption has played a role in their suicide?

☐ Yes

☐ No

☐ I don't know

14.4 If yes, would you like to provide more details? ...

15. ADDICTION

15.1 Did [....] have any addictions at the time of the suicide?

☐ Yes

☐ No

☐ I don't know

15.1.1 If yes, what addiction did [...] have? (multiple answers possible)

☐ Smoking

☐ Alcohol

☐ Drugs, namely ...

☐ Gambling

☐ Gaming/internet

☐ Pornography

☐ Other, namely ...

☐ No

15.2 Do you think an addiction played a role in the suicide?

 Yes

 No

 I don't know

15.3 If yes, would you like to elaborate? ...

16. RELIGION

16.1 Was [....] brought up from a religious background?

 Yes

 No

 I don't know

16.1.1 Had [...] themselves adopted a religion? If yes, what religion did [....] adopt? ...

16.2 Do you think the religion or spiritual beliefs of [....] played a role in the suicide?

 Yes

 No

 I don't know

16.3 If yes, would you like to elaborate? ...

17. DISCRIMINATION

17.1 Did [....] ever feel unfairly treated by other people, for example, because of his/her skin color, sexual preference, religion, or disability? This is referred to as discrimination.

☐ Yes

☐ No

☐ I don't know

17.2 Has [....] experienced any form of discrimination that may have played a role in the suicide?

☐ Yes

☐ No

☐ I don't know

17.3 If yes, would you like to elaborate? ...

18. GENDER IDENTITY AND SEXUAL ORIENTATION

18.1 Did [....] feel attracted to men or women? Or both men and women. In other words, what was the sexual orientation of [....]?

☐ [....] was a man and was attracted to women, heterosexual

☐ [....] was a woman and was attracted to men, heterosexual

☐ [....] was a man and was attracted to men, homosexual

☐ [....] was a woman and was attracted to women, lesbian

☐ [....] was attracted to both women and men, bisexual

☐ Other, namely ...

□ I don't know

18.2 Did [....] experience problems related to their sexual orientation?

□ Yes

□ No

□ I don't know

18.2.1 If yes, what problems were they? ... (from oneself, surroundings)

18.3 Some people have the body of a woman but feel like a man. Or vice versa. This is called transgender. There are also people who have male and female physical characteristics. This is called intersexuality. Yet others feel that they are neither a man nor a woman. This is called non-binary. Was this the case for [....]?

□ Yes, [....] was transgender

□ Yes, [....] was intersexual

□ Yes, [....] was non-binary

□ No

□ I don't know

18.4 Did [....] experience problems related to gender identity?

□ Yes

□ No

□ I don't know

18.4.1 If yes, what problems were they? ... (from oneself, surroundings)

18.5 Do you think problems with gender identity or sexual preference influenced the suicide?

□ Yes

□ No

□ I don't know

□ [....] did not experience problems with gender identity or sexual preference

18.6 If yes, would you like to elaborate? ...

19.Questions about depressed feelings (PHQ-9)

Standard questionnaire (Dutch version, English may be available but we have not translated this).

20.POSTVENTION AND FOLLOW-UP CARE

20.1 Did the funeral director offer you or other bereaved of [...] the opportunity to get in touch with the Dutch Railways Postvention Team?

20.1.1 If yes, did you use this postvention? ...

20.2 If yes, what was your experience with the Dutch Railways Postvention Team? ...

20.3 Have you received any other form of postvention or follow-up care after the suicide of [...]?

20.3.1 What were your experiences with it? ...

21. Reflections and missed topics

21.1 Are there any other things you would like to express that have not been addressed but you feel are important to mention?

21.2 What is your experience of the interview? Do you have feedback that we may incorporate in the future?

Thank the interviewee for their time. Make an appointment to check-in a week after participation. Discuss if there is a potential secondary informant.
